# Genomic Prediction Ability Using Linear and Threshold Approaches for Different Stayability Definitions in Nellore Females

**DOI:** 10.1111/jbg.70033

**Published:** 2025-11-27

**Authors:** Letícia Silva Pereira, Larissa Bordin Temp, Eduarda da Silva Oliveira, Jorge Hidalgo, Cláudio Ulhoa Magnabosco, Fernando Baldi

**Affiliations:** ^1^ National Association of Breeders and Researchers (ANCP) Ribeirão Preto São Paulo Brazil; ^2^ School of Agriculture and Veterinary Science São Paulo State University Jaboticabal São Paulo Brazil; ^3^ Department of Animal and Dairy Science University of Georgia Athens Georgia USA; ^4^ Embrapa Cerrados Brasília Federal District Brazil

**Keywords:** fertility, genomic selection, liability scale, longevity

## Abstract

Enhancing female longevity through regular calvings improves herd replacement rates and reduces the costs associated with replacing low reproductive efficiency animals. Stayability (STAY), defined as a cow's ability to remain productive in the herd, is a categorical trait that challenges traditional genetic evaluation due to its non‐normal distribution. This study aimed to estimate genomic predictions for different STAY definitions—based on the number of calvings at specific ages in Nellore females—and to compare the predictive ability of linear and threshold models using the linear regression (LR) method. Phenotypic and genotypic data from 187 herds provided by the Nellore Brazil breeding program (ANCP, Ribeirão Preto, Brazil) were used. Four STAY definitions (STAY48‐2, STAY48‐3, STAY54‐2, STAY54‐3, STAY72‐3) were evaluated. Genomic estimated breeding values (GEBV) were obtained using univariate linear and threshold models implemented in the BLUPF90 software family. Variance components were transformed from liability to observed scale. Heritability estimates ranged from 0.16 to 0.22 on the liability scale and 0.07 to 0.09 on the observed scale. Threshold models showed superior predictive ability compared to linear models, with higher accuracies (0.531 to 0.698 vs. 0.451 to 0.532), lower bias (−0.0004 to 0.008 vs. 0.027 to 0.096) and dispersion values closer to the ideal (0.932 to 1.000 vs. 0.811 to 0.848). Among the definitions, STAY48 with at least two or three calvings demonstrated the most consistent performance, representing a promising criterion for genetic evaluation in Nellore cattle.

## 1 Introduction

An efficient reproductive lifespan of cows is essential for the success of beef cattle production systems. Regardless of the production system adopted, reproductive traits are paramount in defining selection criteria (Phocas et al. 1998; Brumatti et al. 2011). Stayability (STAY) is a trait that measures a cow's ability to remain in the herd as a function of its productivity, therefore, it is an economically relevant trait in livestock production systems (Portes et al. 2021). Increased female longevity, with regular calvings, can optimise herd replacement rates, which may help reduce costs associated with replacing animals with low reproductive efficiency (Santana et al. 2013). In general, STAY is measured late in the animal's productive life, only in females and exhibits low heritability estimates. These factors can hinder direct selection for this trait, as they tend to increase the generation interval and reduce genetic gain per year (Teixeira et al. 2017; Ramos et al. 2021; Silva et al. 2024).

In selection herds, the implementation of reproductive technologies and genomics has reduced the age at first calving and increased the voluntary culling rate, thereby decreasing the average age of the herd, with a consequent reduction in generation interval (GI). In *Zebu* breeds like Nellore, females are raised in extensive systems in the tropics, where they are subjected to an environment with seasonal feed resources, with STAY being a measure of productivity over several reproductive cycles. Therefore, it is a trait that reflects not only reproductive efficiency but also the ability to adapt to environmental and nutritional conditions, as well as maternal ability.

With the intense selection for sexual precocity, especially in the last 14 years in the Nellore breed, that is, with females challenged between 10 and 14 months of age (Pereira et al. 2025), those that manage to conceive, calving, wean a calf and conceive again at regular reproductive intervals make their longevity an even more effective measure of the functional reproductive longevity of these females in real production environments. These changes in herd structure have necessitated a reevaluation of the selection criteria for productive longevity in females challenged at an early age in selection herds, such as those involved in employment. Earlier definitions of the trait (such as at 48 or 54 months of age) are being replaced by traditional definitions later, like those based on permanence up to 72 months.

As a categorical trait (success or failure), STAY does not follow a normal distribution, which increases the complexity of genetic evaluation models (Cavani et al. 2015; Costa et al. 2019). In this context, genetic values are predicted using threshold models, which assume the existence of an underlying standard variable (liability) that is presumed to have a joint distribution with the observed binary phenotypes (Hidalgo, Tsuruta, et al. 2024). Threshold models require a nonlinear system of equations imposing challenges for convergence and computing time. These challenges are lessened in linear models, which are favoured as well because in practice the advantages provided by threshold models over linear models are minimal for raking purposes. Cappelloni et al. (2022), estimated variance components and genetic values for traits in different pig breeds, comparing threshold and linear models. Their findings indicated that both models provided similar estimates, but the linear model was recommended due to its greater practicality and computational efficiency.

Misztal et al. (1989) identified significant differences in computational cost between threshold and linear models, estimating an increase of approximately five times in computational time for the threshold models. Similarly, Weller et al. (1988) and Clutter et al. (1989) reported an increase of about three times the time required to run threshold models compared to linear models. Supporting these findings, Hidalgo, Misztal, et al. (2024) also highlighted that the convergence time for threshold models was approximately five times longer than that of linear models. The linear models offer a robust approximation, and previous studies in the literature (Weller et al. 1988; Heringstad et al. 2003; Koeck et al. 2010) have reported highly correlated estimated breeding values (EBVs) between linear and threshold models (Hidalgo, Misztal, et al. 2024).

Model validation for categorical traits presents challenges, especially in the context of genetic prediction. The linear regression (LR) method has been proposed as a valuable tool for evaluating the performance of these models and estimating potential improvements in predictive accuracy (Bermann et al. 2021). Given this context, in the present study we aimed to estimate genomic predictions for different STAY definitions (considering varying calvings in Nellore females) and to compare, using the LR method, the predictive ability achieved using linear and threshold models. Considering that STAY is a categorical trait, exploring different statistical approaches becomes essential to ensure more reliable estimates.

## 2 Material and Methods

### 2.1 Ethics Statement

This study was exempt from evaluation by the Animal Ethics Committee (CEUA), as established by Law No. 11,794 of 08/10/2008 and Normative Resolution No. 51 of 05/19/2021 from the National Council for Animal Experimentation Control (CONCEA) because the data was obtained from an existing database.

### 2.2 Phenotypes and Quality Control

The phenotypic and genotypic information was provided by the Nellore Brazil breeding program, coordinated by the National Association of Breeders and Researchers (ANCP, Ribeirão Preto, Brazil). The animals belonged to 187 farms in Brazil's central‐west, southeast, northeast and northern regions. The heifers were exposed to reproduction at 10–14 months of age, and the mating season lasted 3–4 months.

#### 2.2.1 Longevity

Stayability (STAY, %) was considered in this study. To calculate traditional stayability, all females in the database were used, regardless of whether they had been challenged between 10 and 14 months of age or not. To calculate the new stayability approaches in early females, only challenged females were used. For STAY, females born between 2000 and 2019 were classified according to the number of calvings at different ages, as described below (Pereira et al. 2025):

- STAY48‐2: Challenged females that produced two calves by 48 months of age received a score of 2 (success), while those that did not meet the previous criterion received a score of 1 (failure).
- STAY48‐3: Challenged females that produced three calves by 48 months of age received a score of 2 (success), while those that did not meet the previous criterion received a score of 1 (failure).
- STAY54‐2: Challenged females that produced two calves by 54 months of age received a score of 2 (success), while those that did not meet the previous criterion received a score of 1 (failure).
- STAY54‐3: Challenged females that produced 3 calves by 54 months of age received a score of 2 (success), while those that did not meet the previous criterion received a score of 1 (failure).
- STAY72‐3: Challenged females that produced 3 calves by 72 months of age were given a score of 2 (success), while those that did not meet the above criterion were given a score of 1 (failure).

The relationship matrix contained 720,239 animals born from 1990 to 2019 with an average inbreeding coefficient of 0.01% and the percentage of inbred individuals being 0.02%. Contemporary groups (CG) were defined as the combination of farm, management group, year and birth season (dry season from April to September and the rainy season from October to March). The data structure and descriptive statistics for different STAY definitions are presented in Table 1.

**TABLE 1:** Number of records (*N*) and contemporary groups (CG), mean (SD) and percentage (%) of animals within each category of several stayability definitions in Nellore cattle.

Trait	*N*	CG	Mean (SD)	Success (2) %	Failure (1) %
STAY48‐2	182,064	2741	1.14 (0.35)	26,152 (14.36%)	155,912 (85.24%)
STAY48‐3	182,064	2741	1.13 (0.33)	23,685 (13.01%)	158,379 (86.99%)
STAY54‐2	181,613	2717	1.11 (0.31)	20,865 (11.49%)	160,748 (88.51%)
STAY54‐3	181,613	2717	1.11 (0.31)	20,040 (11.03%)	161,573 (88.97%)
STAY72	646,918	15,967	1.38 (0.48)	246,079 (38.03%)	400,839 (61.97%)

*Note:* STAY48‐2: female's ability to remain in the herd up to 48 months of age, calving to at least two times; STAY48‐3: female's ability to remain in the herd up to 48 months of age, calving to at least three times, STAY54‐2: female's ability to remain in the herd up to 54 months of age, calving to at least two times; STAY54‐3: female's ability to remain in the herd up to 54 months of age, calving to at least three times; STAY72‐3: female's ability to remain in the herd up to 72 months of age, calving to at least three times.

### 2.3 Genotypes and Quality Control

A total of 402,130 animals were genotyped with the low‐density panel (Clarifide Nellore 3.0) containing more than 54,000 markers. The genotype quality control was performed using the PreGSF90 program (Aguilar et al. 2014). Data quality control removed: samples and markers with a call rate < 0.90, markers located on sex and mitochondrial chromosomes, markers with mendelian conflicts > 1%, minor allele frequency < 0.05, markers with redundant position and markers with deviation from Hardy Weinberg Equilibrium (*p* < 10^−10^). After quality control 42,349 SNPs were retained in the database for analysis.

### 2.4 Statistical Model and Genetic Analyses

Univariate statistical analyses were performed to predict breeding values by fitting threshold and linear models, using single‐step genomic best linear unbiased prediction (ssGBLUP). Computations were done using CBLUP90IOD2OMP1 (v. 3.38) for threshold models and BLUP90IOD3 (v. 3.138) for linear models (Misztal et al. 2014). Variance components for STAY were transformed from liability to observed scale to obtain the genomic estimated breeding values (GEBV) using linear models. This conversion process relied on the formula originally developed by Robertson (refer to Dempster and Lerner (1950)), which was later expanded to multiple‐categorical traits by Gianola (1979).

The linear model, under matrix notation, can be written as:

y=Xβ+Zu+e,

where: y is the vector of phenotypic observations; β is the vector of fixed effects (CG); u is the vector of random additive animal effects; e is the vector of random residuals; X is the incidence matrix relating the effects in β to observations in y; and Z is the incidence matrix relating the levels in u to observations in y.

Under multivariate normality assumption, the (co)variance matrix for the random effects is given by:

$$\mathrm{Var}\left[\begin{array}{c} u\\ e \end{array}\right]=\left[\begin{array}{cc} H\sigma_{u}^{2} & 0\\ 0 & I\sigma_{e}^{2} \end{array}\right],$$

where: **H** is a joint covariance matrix for non‐genotyped and genotyped animals (Legarra et al. 2009); I is an identity matrix of proper order; $\sigma_{u}^{2}$ is the additive genetic variance for the trait being analysed; and $\sigma_{e}^{2}$ is the residual variance.

The inverse of **H** was computed as in Aguilar et al. (2010):

$$H^{-1}=A^{-1}+\left[\begin{array}{cc} 0 & 0\\ 0 & G^{-1}-A_{22}^{-1} \end{array}\right],$$

where **A** ^**−1**^ is the inverse of pedigree‐based relationship matrix for all animals included in the pedigree, $A_{22}^{-1}$ is the inverse of the pedigree‐based relationship matrix for the genotyped animals. The genomic relationship matrix (**G**) was constructed based on the first method proposed by VanRaden (2008) as $G=ZZ^{\prime }$, in which $Z=\left(M-P\right)/\left[2\sum_{j=1}^{n}p_{j}\left(1-p_{j}\right)\right]$ with M being a matrix with genotypes coded as {0, 1, 2, 5 (missing)}, number of rows equal to the number of genotyped animals and number of columns equal to the number of SNP. P is a matrix with twice the observed frequency of the second allele p at locus j ($p_{j}$) in the entire genotype file. It incorporates a blending approach in which 5% of the pedigree‐based relationship matrix for genotyped animals (**A**_22_) is included to mitigate singularity issues.

Additionally, **G** was rescaled to align with **A**_22_ by adjusting its diagonal and off‐diagonal elements (VanRaden 2008). The GEBV was calculated using the algorithm for proven and young (APY; Misztal et al. 2014) to ensure computational feasibility and efficiency. The APY method relies on a recursive approach applied to a subset of animals for the inversion of the **G** matrix, which is divided into core (c: 20,105) and non‐core (n: 382,025) animals. The number of core animals is selected to approximate the dimensionality of **G** or the number of effective single nucleotide polymorphisms (SNPs) (Misztal et al. 2014).

A threshold model was implemented via Bayesian inference, considering CG as a systematic effect and animal additive as a random effect. Under matrix notation, the model for a latent variable (liability) is: l=Xβ+Zu+e, where: l is the vector of liabilities; β, u, e, X and Z, were defined previously. Given β and u, the elements of l are conditionally independent and distributed as: $\left.l\right|\beta ,u,\sigma_{u}^{2},\sigma_{e}^{2}\sim \mathrm{MVN}\left\{\mathrm{X\beta}+\mathrm{Zu},I\sigma_{e}^{2}\right\}$. It was also assumed that: $\left.\beta \right|{I,\sigma }_{\beta }^{2}\sim \mathrm{MVN}\left\{0,I\sigma_{\beta }^{2}\right\}$; $\left.u\right|{H,\sigma }_{u}^{2}\sim \mathrm{MVN}\left\{0,H\sigma_{u}^{2}\right\}$, where H is, the relationship matrix; and $\left.e\right|{I,\sigma }_{e}^{2}\sim \mathrm{MVN}\left\{0,I\sigma_{e}^{2}\right\}$, where I is an identity matrix of proper order; $\left.\sigma_{u}^{2}\right|\nu_{u}\sim \chi^{-2}\left(\nu_{u},S_{u}^{2}\right)$ and $\left.\sigma_{e}^{2}\right|\nu_{e}\sim \chi^{-2}\left(\nu_{e},S_{e}^{2}\right)$, being $\chi^{-2}$ an inverted chi‐square distribution with ν degrees of belief and S scale matrix.

### 2.5 Variance Components and Heritability Estimates

The variance components were estimated via Gibbs sampling implemented in the GIBBSf90+ software (Misztal et al. 2014) using the default prior for random and systematic effects (i.e., Gaussian prior for random effects and flat for systematic). In the Gibbs Sampling implementation, 300,000 iterations were employed, with an initial burn‐in of 100,000 iterations and a sampling interval of 100 iterations (thin). The POSTGIBBSF90 software (Misztal et al. 2002) was used for posterior inference. Convergence was assessed through visual inspection of the generated Markov chains and by applying convergence diagnostics as follows: (1) Geweke's test (Geweke 1992), which compares the initial and final segments of the chain to detect convergence failures.

Rejection of the null hypothesis in this test indicates convergence, with probabilities below 0.05 providing evidence against chain convergence (Buzanskas et al. 2010); (2) Heidelberg and Welch test (Welch and Heidelberger 1983) was utilised, employing Cramer–von Mises statistics to evaluate the null hypothesis of stationarity in the generated samples and (3) Raftery and Lewis test (Raftery and Lewis 1992). All methods are implemented in the ‘boa’ R package (Smith 2007).

Variance components were estimated on the liability scale and then transformed to the observed scale to implement a linear–linear model for predicting breeding values. The transformation was based on the following formula (Dempster and Lerner 1950), derived for binary traits and described by Hidalgo, Tsuruta, et al. (2024): $h_{o}^{2}=z^{2}h_{l}^{2}/\propto \left(1-\propto \right)$, where $h_{o}^{2}$ is the additive heritability on the observed scale, $z^{2}$ is the height of the ordinate of the standard normal probability density function at the point corresponding to the threshold between categories, estimated from the incidence of success (*α*) of the trait, $h_{l}^{2}$ is the heritability on the liability scale. Also, the formula allowed to obtain the additive ($\sigma_{u}^{2}=z^{2}h_{l}^{2}$) and phenotypic ($\sigma_{p}^{2}=\propto \left(1-\propto \right)$) variances on the observed scale. The residual variance in the observed scale was obtained by $\sigma_{e}^{2}=\sigma_{p}^{2}-\left(\sigma_{u}^{2}+\sigma_{\mathrm{pe}}^{2}+\sigma_{\mathrm{cg}}^{2}\right)$, where $\sigma_{\mathrm{pe}}^{2}$ and $\sigma_{\mathrm{cg}}^{2}$ are the random effects of the permanent environment and the contemporary group of the cow, in our case zero.

### 2.6 Evaluation of Model Performance: Prediction Accuracy, Bias, Dispersion and Correlation

Scenarios were compared using the LR (Legarra and Reverter 2018) method. The whole dataset (represented by subscript w) included all sources of information for all animals (Table 2). The partial dataset was created by removing the last 2 years of data. This was done to ensure an enough number of focal animals for validation. Focal animals were young, genotyped females without their own (or progeny) records in the partial dataset.

**TABLE 2:** Number of records in the whole and partial datasets and validation animals used in the linear regression method for the different scenarios for the longevity trait in the Nellore breed.

	STAY 48	STAY 54	STAY72
Whole	182,064	181,613	646,918
Partial	172,852	172,852	610,623
Validation^a^	9212	8761	10,704

^a^ Young animals with genotypes and phenotypes.

The validation statistics were accuracy (acc), bias (δ), dispersion (b_1_) and correlation (corr), computed as follows:

$$\mathrm{acc}=\sqrt{\frac{\mathrm{cov}\left({\hat{u}}_{w},{\hat{u}}_{p}\right)}{\left(1-\bar{F}\right){\hat{\sigma }}_{u}^{2}}},$$

$$\delta =\frac{{\bar{\hat{u}}}_{p}-{\bar{\hat{u}}}_{w}}{{\hat{\sigma }}_{u}},$$

$$b_{1}=\frac{\mathrm{cov}\left({\hat{u}}_{w},{\hat{u}}_{p}\right)}{\mathrm{var}\left({\hat{u}}_{p}\right)},$$

$$\text{corr}=\frac{\mathrm{cov}\left({\hat{u}}_{w},{\hat{u}}_{p}\right)}{\sqrt{\mathrm{var}\left({\hat{u}}_{w}\right)\mathrm{var}\left({\hat{u}}_{p}\right)}},$$

where: $\mathrm{cov}\left(\cdot \right)$ is the sample covariance; ${\hat{u}}_{w}$ is the vector of GEBV from the whole dataset; ${\hat{u}}_{p}$ is the vector of GEBV from the partial dataset; $\bar{F}$ is the average pedigree‐inbreeding coefficient among focal animals; ${\hat{\sigma }}_{u}^{2}$ is the estimated additive genetic variance; ${\bar{\hat{u}}}_{p}$ is the average of ${\hat{u}}_{p}$ (likewise for ${\bar{\hat{u}}}_{w}$); and $\mathrm{var}\left(\cdot \right)$ is the sample variance.

## 3 Results and Discussion

The heritability estimates for the different categories of the STAY trait were low (Table 3), ranging from 0.16 to 0.22 on the liability scale. When transformed to the observed scale, the heritability estimates ranged from 0.07 to 0.09 and were lower than those obtained on the liability scale.

**TABLE 3:** Posterior means and high probability density (HPD^a^) for direct additive genetic ($\sigma_{a}^{2}$), residual ($\sigma_{e}^{2}$) variances and heritability (SD, standard deviation) for different categories of stayability in Nellore cattle.

Trait	$\sigma_{a}^{2}$	$\sigma_{a}^{2}$ ^b^	$\sigma_{e}^{2}$	$\sigma_{e}^{2}$ ^b^	h^2^	h^2^ ^b^	±SD	HPD
STAY48‐2	0.24	0.010	1.01	0.113	0.20	0.08	0.01	0.16 to 0.22
STAY48‐3	0.26	0.009	1.00	0.104	0.20	0.08	0.01	0.17 to 0.23
STAY54‐2	0.27	0.008	1.00	0.094	0.22	0.07	0.02	0.18 to 0.24
STAY54‐3	0.28	0.008	1.00	0.090	0.22	0.07	0.02	0.19 to 0.25
STAY72‐3	0.19	0.023	1.00	0.213	0.16	0.09	0.01	0.14 to 0.18

*Note:* STAY48‐2: female's ability to remain in the herd up to 48 months of age, calving to at least two times; STAY48‐3: female's ability to remain in the herd up to 48 months of age, calving to at least three times; STAY54‐2: female's ability to remain in the herd up to 54 months of age, calving to at least two times; STAY54‐3: female's ability to remain in the herd up to 54 months of age, calving to at least three times; and STAY72‐3: female's ability to remain in the herd up to 72 months of age, calving to at least three times.

^a^ Credibility intervals at 95%.

^b^ Observable scale.

The genomic prediction parameters for the validation animals for each trait are presented in Table 4. Ramirez‐Valverde et al. (2001) and Koeck et al. (2010) reported a high correlation (≥ 0.96) between EBVs of linear and threshold models. Varona et al. (1999) found no advantage of the threshold model over the linear model for calving ease. Similar results between the two models were also observed by Weller et al. (1988) in evaluations of calf mortality and dystocia in Holstein cattle, as well as by Matos et al. (1997) in reproductive traits of sheep.

**TABLE 4:** Genomic prediction accuracy, bias, dispersion and correlation of genomic predictions for different stayability definitions in Nellore cattle using threshold (Th) and linear models (Li).

Trait	Accuracy		Bias		Dispersion		Correlation	
	Th	Li	Th	Li	Th	Li	Th	Li
STAY48‐2	0.558	0.503	0.002	0.027	0.997	0.842	0.997	0.986
STAY48‐3	0.531	0.532	0.008	0.028	0.932	0.847	0.955	0.986
STAY54‐2	0.551	0.510	0.135	0.031	0.974	0.848	0.892	0.988
STAY54‐3	0.567	0.514	−0.0004	0.031	1.000	0.848	1.000	0.988
STAY72‐3	0.698	0.451	−0.017	0.096	0.977	0.811	0.995	0.959

*Note:* STAY48‐2: female's ability to remain in the herd up to 48 months of age, calving to at least two times; STAY48‐3: female's ability to remain in the herd up to 48 months of age, calving to at least three times. STAY54‐2: female's ability to remain in the herd up to 54 months of age, calving to at least two times; STAY54‐3: female's ability to remain in the herd up to 54 months of age, calving to at least three times and STAY72‐3: female's ability to remain in the herd up to 72 months of age, calving to at least three times.

The accuracy values obtained for the different STAY definitions were higher in the threshold model, with values ranging from 0.531 to 0.698, compared to the linear model, whose values ranged from 0.451 to 0.532. Ramirez‐Valverde et al. (2001), when analysing the trait of calving difficulty in beef cattle, reported that the accuracy of the threshold model EBVs was 10% higher than that of linear model EBVs, in line with our results as we found improvements in accuracy from 10% (0.503 vs. 0.558; STAY48‐2) and up to 35% (0.451 vs. 0.698; STAY72‐3). Our results indicate that the model based on the observable scale led to a reduction in accuracy, while increasing bias compared to models operating on the liability scale (Table 4). As an illustration, for STAY72‐3 the bias using a threshold model was −0.017, whereas it was 0.096 when using a linear model.

Considering the categorical nature of STAY, the application of the threshold model is theoretically the most appropriate choice, as proposed by Gianola (1982). However, from a practical standpoint, the linear model offers greater applicability (Varona et al. 1999; Silvestre et al. 2019). Another important factor to consider is the increased computational effort required by the threshold model. Although advantages of the threshold model over linear models have been reported in simulated data (Meijering and Gianola 1985), studies using real data have yielded variable results (Silvestre et al. 2019; Matos et al. 1997).

Conceptually, the greater the data availability, the more robust the approximation of linear models can become, even with deviations from normality (Matos et al. 1997). However, when considering the evaluation of reproduction traits, such as fertility expressed as a binary trait, litter size with four categories and ovulation rate with five categories in a small population of sheep, Matos et al. (1997) did not identify superiority of the threshold model in relation to the linear model, since the predictive abilities were similar: 0.21 for the linear model and 0.20 for the threshold model. Casellas et al. (2007) also reported minimal differences in the predictive ability of linear and threshold models in a similar study for the genetic evaluation of litter size and days until parturition in a sheep population. Carlén et al. (2006) reported in a simulation study that there were minimal gains in replacing the linear model with a threshold model or with survival analysis in the genetic evaluation of mastitis in dairy cattle.

Regarding bias, the lowest values were generally observed for the threshold model, while the linear model yielded slightly higher bias. The results indicated that the bias estimates obtained with the threshold model were close to zero (ranging from −0.0004 to 0.008), except for STAY54‐2, which presented a higher bias value (0.135). For the linear model, bias values were slightly higher, ranging from 0.031 to 0.096, except for STAY54‐2, where the bias (0.031) was lower than that observed with the threshold model. Generally, more biased predictions were associated with lower accuracy.

The results indicated that the dispersion values for the linear model differed from the expected value of 1 (0.811 to 0.827). In contrast, dispersion values observed in the threshold model were closer to or at the ideal value (0.932 to 1.00). The STAY is defined as the probability of a female remaining in the herd until a certain age, reflecting her capacity for reproductive efficiency and is relevant for the new classifications proposed for those early females, which, according to the descriptive table, can observe a similar success rate. This pattern is due to the low reconception rate in the second reproductive season, as STAY is a trait with a high influence from the environmental component (i.e., low heritability) and high culling of animals for different reasons, such as being empty at the end of the reproductive season.

For example, for females that had two and three calvings by 48 months of age, the success rate was 14.36% and 13.01%, respectively. The success rate was even lower for females that had two and three calvings by 54 months of age, 11.49% and 11.03%, respectively. The latter can be a possible explanation for STAY54‐2 presenting higher biases and lower genetic correlations of genetic values in both models; SATY72‐3, for example, showed a success rate of 38.03% and better LR statistics, that is, higher accuracy and correlations, and lower bias and dispersion.

The imbalance in class frequencies can directly influence the predictive ability of the models, especially when using the linear model. In low success rate scenarios such as the 48‐ and 54‐month classifications, where there are high discarding rates, linear models tend to underestimate the breeding values, as demonstrated in the results found in the bias and dispersion values. Even though linear models do not consider the binary nature of binary traits, the correlation of genetic values between the linear and threshold models followed the same trend, approximately close to 1, except for STAY54‐2, where the correlation values of the threshold and linear models were 0.892 and 0.988, respectively. In practical terms, the reclassification of animals in both models would be minimal.

According to Kizilkaya et al. (2014), in certain situations, linear models can present similar efficiency to threshold models. This behaviour is expected as the number of categories increases and approaches normality. The least favourable situation is for binary data, as predictive capacity declines as the number of categories, as reported by the same authors. In their results, these authors also reported that the efficiency of the analysis of binomial data transformed into continuous data also tends to reduce as the observed frequency moves away from 0.5 (i.e., with more imbalance between categories).

Although the threshold model is theoretically the most appropriate approach for analysing categorical traits, practical results do not always conform to this expectation. Working with longevity in the Italian Jersey breed, Fabris et al. (2025) demonstrated that EBVs can be estimated using a linear model with nearly equivalent performance, but with less computational effort and greater routine feasibility. Previous studies have also reported contrasting outcomes, showing cases in which threshold models performed better (Colonia et al. 2022; Camargo Júnior et al. 2025), worse (Carlén et al. 2006) or comparably to linear approaches (Campos et al. 2018).

## 4 Conclusion

Overall, converting the trait to a linear model resulted in increased bias and reduced accuracy of genomic predictions compared to the threshold model. The threshold model demonstrated superior predictive ability (in terms of bias, dispersion and accuracy), particularly for the earlier definitions (STAY48 and STAY54), and is therefore the most suitable approach for genomic prediction of stayability in this dataset.

Among the different STAY definitions evaluated for early‐challenged females, the STAY48 definition, requiring at least two or three calvings, stood out for its consistent and satisfactory performance in both models, representing a promising alternative for use in genetic evaluations. On the other hand, although the prediction accuracies of the earlier definitions are slightly lower, they represent more efficient traits for guiding culling and replacement decisions in breeding programs, as they facilitate informed decision‐making, allow for greater selection intensity and consequently accelerate genetic and productive progress in the herd.

It is important to acknowledge the trade‐off between predictive accuracy, heritability and the age at which the phenotype is measured for selection purposes. Although the latter definition, STAY72‐3 (measured at 6 years of age), presented slightly higher accuracy values and lower bias in the threshold model, this category showed lower heritability (0.16) compared to the earlier definitions, whose heritabilities ranged between 0.20 and 0.22. Thus, its use as a selection criterion tends to reduce the expected response to selection per unit of time, since the decision is postponed to later stages of the productive life. Therefore, the use of the definitions measured at 48 or 54 months is more appropriate, depending on the specific breeding objective of the production system.

## Funding

The authors have nothing to report.

## Conflicts of Interest

The authors declare no conflicts of interest.

## Data Availability

The data that support the findings of this study are available on request from the corresponding author. The data are not publicly available due to privacy or ethical restrictions.
